# Toxoplasma gondii Dissemination in the Brain Is Facilitated by Infiltrating Peripheral Immune Cells

**DOI:** 10.1128/mbio.02838-22

**Published:** 2022-11-29

**Authors:** Christine A. Schneider, Dario X. Figueroa Velez, Stephanie B. Orchanian, Lindsey A. Shallberg, Dritan Agalliu, Christopher A. Hunter, Sunil P. Gandhi, Melissa B. Lodoen

**Affiliations:** a University of California Irvine, Department of Molecular Biology and Biochemistry, Irvine, California, USA; b University of California Irvine, Institute for Immunology, Irvine, California, USA; c University of California Irvine, Department of Neurobiology and Behavior, Irvine, California, USA; d University of California Irvine, Center for the Neurobiology of Learning and Memory, Irvine, California, USA; e University of Pennsylvania School of Veterinary Medicine, Department of Pathobiology, Philadelphia, Pennsylvania, USA; f Columbia University Irving Medical Center, Department of Pathology and Cell Biology, New York, New York, USA; g Columbia University Irving Medical Center, Department of Neurology, New York, New York, USA; Albert Einstein College of Medicine

**Keywords:** *Toxoplasma*, central nervous system infections, host-pathogen interactions, immunity, parasite, pathogenesis

## Abstract

Despite recent advances in our understanding of pathogenic access to the central nervous system (CNS), the mechanisms by which intracellular pathogens disseminate within the dense cellular network of neural tissue remain poorly understood. To address this issue, longitudinal analysis of Toxoplasma gondii dissemination in the brain was conducted using 2-photon imaging through a cranial window in living mice that transgenically express enhanced green fluorescent protein (eGFP)-claudin-5. Extracellular T. gondii parasites were observed migrating slowly (1.37 ± 1.28 μm/min) and with low displacement within the brain. In contrast, a population of highly motile infected cells transported vacuoles of T. gondii significantly faster (6.30 ± 3.09 μm/min) and with a higher displacement than free parasites. Detailed analysis of microglial dynamics using CX3CR1-GFP mice revealed that T. gondii*-*infected microglia remained stationary, and infection did not increase the extension/retraction of microglial processes. The role of infiltrating immune cells in shuttling T. gondii was examined by labeling of peripheral hematopoietic cells with anti-CD45 antibody. Infected CD45^+^ cells were found crawling along the CNS vessel walls and trafficked T. gondii within the brain parenchyma at significantly higher speeds (3.35 ± 1.70 μm/min) than extracellular tachyzoites. Collectively, these findings highlight a dual role for immune cells in neuroprotection and in facilitating parasite dissemination within the brain.

## INTRODUCTION

Although infection of the central nervous system (CNS) was once considered a rare event, there is a growing appreciation that myriad pathogens can access and infect the brain (1). To establish CNS infection, neuroinvasive pathogens can breach the blood-brain barrier (BBB) and then disseminate within the brain parenchyma. A variety of mechanisms have been proposed for pathogen spread to the brain, which vary depending on the microbe (bacterial, viral, fungal, or parasitic) and the site of infection (1–3). Toxoplasma gondii is an obligate intracellular neurotropic parasite that is estimated to chronically infect one-third of the global human population, with seroprevalence greater than 70% in some countries (4). T. gondii is an important human pathogen, posing a particular threat to immunocompromised populations, including HIV/AIDS patients and organ transplant recipients (5–7). In the immunocompetent host, acute infection is often asymptomatic, but T. gondii establishes a chronic, lifelong infection in the CNS. Parasite reactivation can occur in individuals during immune suppression and lead to toxoplasmic encephalitis (8).

T. gondii is a foodborne pathogen, and infection typically occurs due to ingestion of food or water contaminated with infectious parasite cysts. During acute infection, the parasites disseminate: after breaching the intestinal epithelium, fast-replicating tachyzoites enter the circulation and spread to distal sites through the bloodstream or lymphatics (9). T. gondii tachyzoites eventually arrive at the BBB, enter the brain, convert to bradyzoites, and establish a chronic infection (10). In the CNS, T. gondii can infect several different brain-resident cells, although the bradyzoite stage of the parasite is most often found encysted within neurons (11–13).

In its infected host, T. gondii may disseminate by its own motility or within infected cells (9). T. gondii is a eukaryotic pathogen that uses its actin-myosin machinery to glide on cellular surfaces and to directly invade host cells (14). Within infected host cells, the parasite forms a vacuole and replicates, eventually leading to cell lysis and the infection of neighboring cells (15). *In vitro*, parasite invasion of motile immune cells increases the motility of these cells compared to that of uninfected cells (16–20). This parasite-induced “hypermotility” has been proposed as a mechanism for facilitating parasite dissemination. As determined by *ex vivo* imaging of mouse peripheral tissues, T. gondii infection increases the motility of infected NK cells in the lymph nodes (21) and infected myeloid cells in the spleen and skin (22). These findings have led to the hypothesis that parasite dissemination may be aided by this induced hypermotility phenotype, but whether parasite-induced hypermotility occurs in the brain remains unknown.

Prior studies indicate that T. gondii enters the brain by direct infection and lysis of the endothelial cells of the BBB (23) or via a myeloid cell “Trojan horse” (24, 25), but little is known about parasite dissemination within the brain. In this study, we used intravital 2-photon time-lapse microscopy through a cranial window in live mice to track the movement of individual extracellular parasites and parasite-infected cells in the brain. The findings reveal that infiltrating immune cells traffic T. gondii within the cerebral blood vessels and within the brain, and that infected cells significantly enhance the dissemination of T. gondii through the brain compared to extracellular parasites migrating on their own. Parasites were found predominantly in infiltrating monocytes and CD8^+^ T cells. We also found that cells infected with T. gondii travel more slowly than motile cells without parasites. Thus, the parasite-induced hypermotility of infected cells reported for peripheral tissues does not appear to occur to a significant degree in the brain. These findings provide novel insights into the dissemination of T. gondii in the brain and reveal unique features of pathogen spread in this organ.

## RESULTS

### Tracking T. gondii motility in the infected brain.

To observe T. gondii motility in the brain during infection, we imaged the parasites in Tie2::eGFP-claudin-5 reporter mice. These mice express a fusion protein of enhanced green fluorescent protein (eGFP) with the tight junction protein claudin-5 under the control of the endothelial cell Tie2 promoter. Claudin-5 is the predominant tight junction-associated protein responsible for a paracellular blood-brain barrier (26, 27). The expression of this fusion protein enables visualization of the blood vessels without obscuring the interior lumen of the vessel and without shadowing effects of deep vessels by those that are more superficial. Since eGFP-claudin-5 mice express transgenic claudin-5 in addition to the endogenous protein (27), we initially confirmed that these mice were comparable to wild-type (WT) C57BL/6 mice in survival, CNS parasite burden, and immune cell recruitment to the brain during T. gondii infection (Fig. S1 and Text S1 in the supplemental material).

10.1128/mbio.02838-22.8 FIG S1 T. gondii infection of eGFP-Claudin-5 mice. Wild-type C57BL/6 mice or eGFP-claudin-5 mice were injected with PBS (mock) or infected with 5 × 10^4^ type II tdTomato-expressing T. gondii tachyzoites. (A) Survival curves of C57BL/6 mice and eGFP-claudin-5 mice. (B) Parasite burden in the brain was determined by quantification of the T. gondii B1 gene in brain homogenates. (C) Infiltrating myeloid cells or lymphocytes were quantified by antibody staining and flow cytometry of brain homogenates from mock- or T. gondii-infected mice. In panel A, *n*_C57BL/6J_ = 8 mice and *n*_eGFP-claudin-5_ = 9 mice from 3 independent experiments. The surviving mice were analyzed for panels B and C. The Mantel-Cox log rank test and the Gehan-Breslow-Wilcoxon test were used for statistical analysis in panel A. Student’s *t* test was used for the statistical analysis in panels B and C to compare C57BL/6J mice to eGFP-claudin-5 mice. n.s., not significant. Download FIG S1, DOCX file, 0.2 MB. Copyright © 2022 Schneider et al. 2022 Schneider et al. https://creativecommons.org/licenses/by/4.0/ This content is distributed under the terms of the Creative Commons Attribution 4.0 International license.

10.1128/mbio.02838-22.7 TEXT S1 Supplementary methods for Fig. S1 and S2. Download Text S1, DOCX file, 0.02 MB. Copyright © 2022 Schneider et al. 2022 Schneider et al. https://creativecommons.org/licenses/by/4.0/ This content is distributed under the terms of the Creative Commons Attribution 4.0 International license.

**FIG 1:**
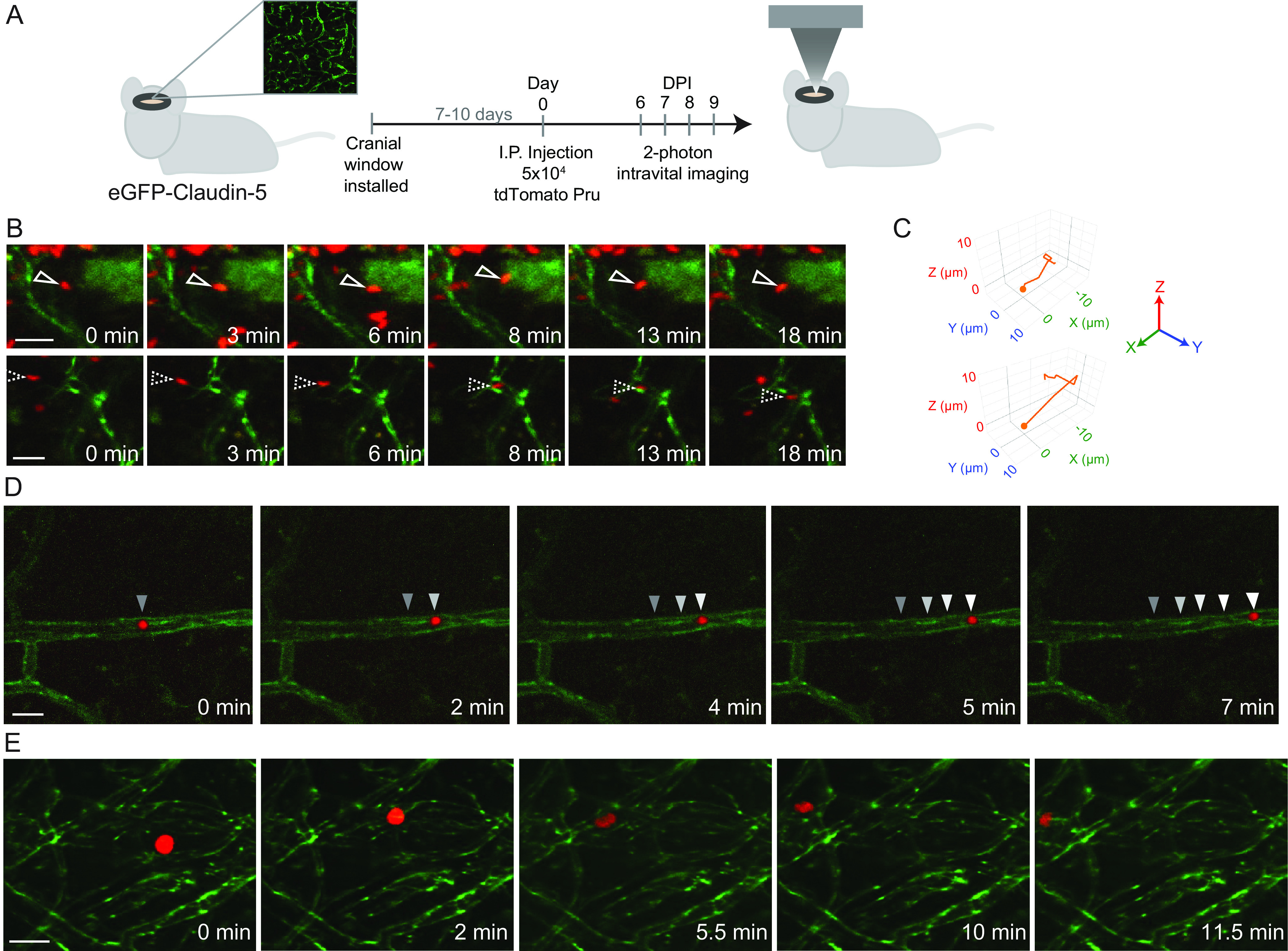
Singlet T. gondii tachyzoites and infected cells with intracellular T. gondii travel through the brain. A cranial window was installed in eGFP-claudin-5 mice, and the mice were infected with 5 × 10^4^ tdTomato-expressing type II T. gondii tachyzoites. Longitudinal time-lapse 2-photon imaging was performed during acute infection in the brain. (A) Schematic of the experimental workflow. (B) Individual singlet parasites were found in clusters, migrating with corkscrew-like motility. eGFP-claudin-5 is shown in green and T. gondii in red. (C) Motility map of the individual tachyzoites shown in panel B in 3D space. (D and E) Parasite vacuoles transported within infected cells in the vessel lumen (D) and through the brain parenchyma (E). Scale bars, 20 μm (B) or 30 μm (D and E). Videos were collected at 7 dpi (D) and 9 dpi (B and E) from 3 different mice.

The eGFP-claudin-5 mice were surgically implanted with a cranial window as previously reported (28) and then intraperitoneally infected with tdTomato-expressing type II T. gondii. Two-photon microscopy imaging was performed from 5 to 9 days postinfection (dpi) (Fig. 1A). During these acute time points, we visualized individual stationary tachyzoites, as well as numerous motile singlet parasites (Fig. 1B and Movie S1). In the brain, singlet parasites exhibited similar motility to the irregular corkscrew-like motility of T. gondii tachyzoites previously described migrating in the mouse earflap (Fig. 1B and C) (29). Interestingly, by 7 dpi, we also observed large vacuoles of T. gondii moving through the cerebral blood vessels and the brain parenchyma. Since the parasitophorous vacuole forms only within host cells and is not known to have inherent motility, we infer that these large vacuoles of parasites were actively transported within infected motile cells. Similar to our previous observations of immune cells rolling at the BBB of T. gondii*-*infected mice (28), we detected infected cells harboring multiple intracellular T. gondii organisms migrating in the vasculature (Fig. 1D and Movie S2). Frequently, we observed parasites being rapidly transported by infected cells outside blood vessels and within the brain parenchyma (Fig. 1E). The vacuoles within infected cells often became highly distorted during transport, with dynamic changes in the configuration of the intracellular parasites (Fig. 1E and Movie S3), suggesting that the infected cells were navigating densely packed tissue.

10.1128/mbio.02838-22.1 MOVIE S1 Motility of singlet tachyzoite in the brain at 9 dpi. eGFP-claudin-5 mice were infected with tdTomato-expressing T. gondii. Two-photon imaging through a cranial window was conducted at 1 frame every 0.5 to 1 min for 18 min. This movie corresponds with the bottom filmstrip in Fig. 1B in the main text. Download Movie S1, AVI file, 0.02 MB. Copyright © 2022 Schneider et al. 2022 Schneider et al. https://creativecommons.org/licenses/by/4.0/ This content is distributed under the terms of the Creative Commons Attribution 4.0 International license.

10.1128/mbio.02838-22.2 MOVIE S2 Motility of an infected cell in a cerebral blood vessel at 7 dpi. eGFP-claudin-5 mice were infected with tdTomato-expressing T. gondii. Two-photon imaging through a cranial window was conducted at 7 dpi. An infected cell with a vacuole of T. gondii was visualized migrating in the lumen of a blood vessel in the brain. Time-lapse imaging was performed at 1 frame every 0.5 to 1 min. Seven minutes of imaging is shown. This movie corresponds with the filmstrip in Fig. 1D. Download Movie S2, AVI file, 0.5 MB. Copyright © 2022 Schneider et al. 2022 Schneider et al. https://creativecommons.org/licenses/by/4.0/ This content is distributed under the terms of the Creative Commons Attribution 4.0 International license.

10.1128/mbio.02838-22.3 MOVIE S3 Motility of a T. gondii*-*infected cell in the brain at 9 dpi. eGFP-claudin-5 mice were infected with tdTomato-expressing T. gondii. Two-photon imaging through a cranial window was conducted at 9 dpi. An infected cell with a vacuole of T. gondii was imaged moving through the brain parenchyma. The cell was imaged for 15.5 min at 1 frame every 0.5 to 1 min. This movie corresponds with the filmstrip in Fig. 1E (filmstrip timestamps of 0 to 11.5 min correspond with 4 to 15.5 min of the video). Download Movie S3, MOV file, 0.1 MB. Copyright © 2022 Schneider et al. 2022 Schneider et al. https://creativecommons.org/licenses/by/4.0/ This content is distributed under the terms of the Creative Commons Attribution 4.0 International license.

### T. gondii infection of motile cells facilitates parasite spread in the brain.

**FIG 2:**
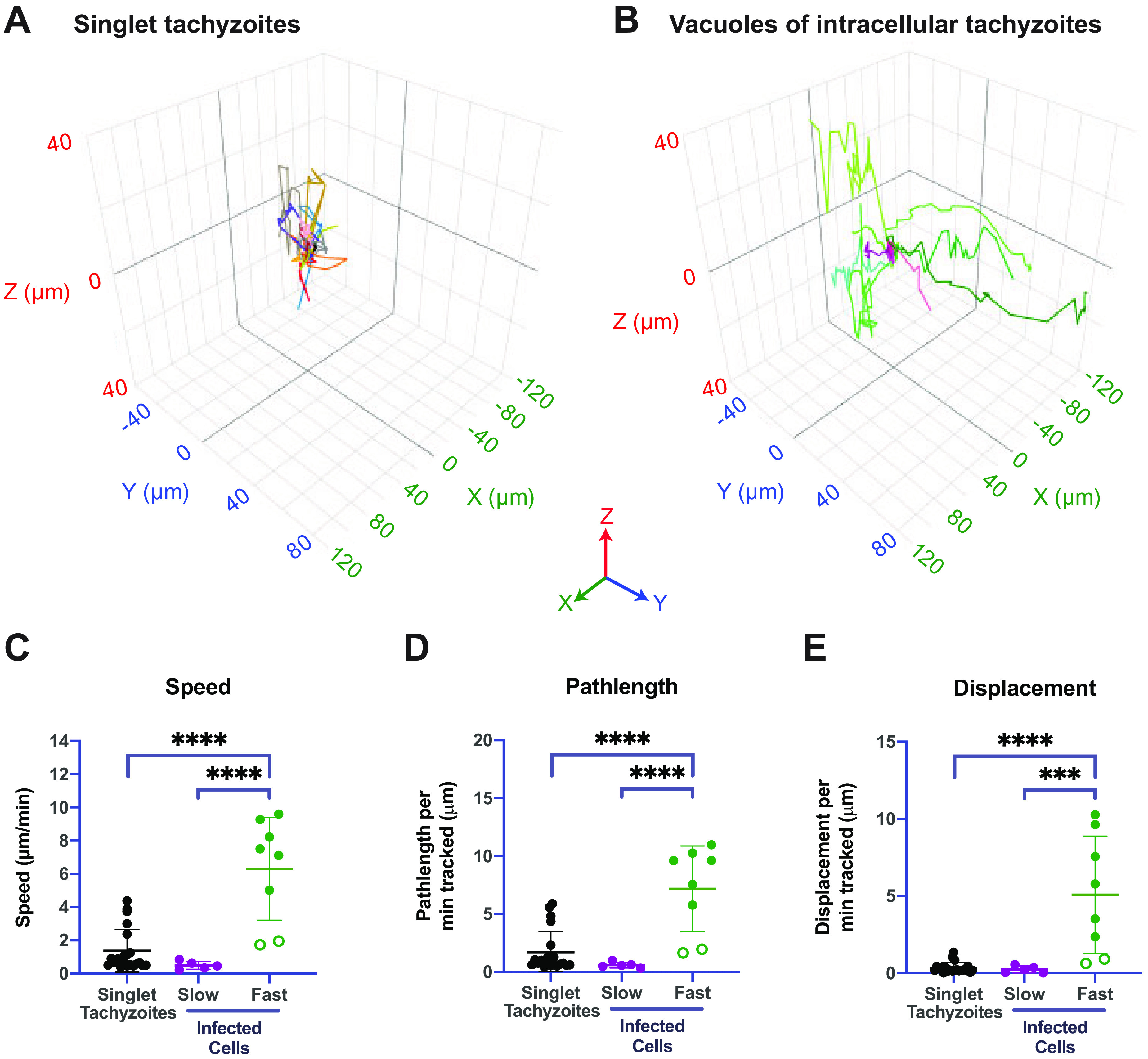
T. gondii-infected cells travel significantly farther and faster than singlet tachyzoites in the brain. Two-photon imaging was conducted through a cranial window in eGFP-claudin-5 mice infected with tdTomato-expressing T. gondii. (A and B) Motility plots of singlet tachyzoites (A) or vacuoles within infected cells (B) that were tracked in 3D by time-lapse 2-photon microscopy. Each trace represents an individual cell, with all traces centered at the origin. (C to E) Average speed (C), path length (D), and displacement (E) of singlet tachyzoites or infected cells. Green closed circles in the fast-moving infected cell category represent cells that exhibited continuous motility, whereas green open circles represent cells that paused at some point during their movement. *n* = 21 tachyzoites from two mice (A and C to E) and *n* = 13 infected cells from two mice (B and C to E). ***, *P* < 0.001; ****, *P* < 0.0001 by analysis of variance (ANOVA). Imaging from 7 to 9 dpi is shown.

There appeared to be markedly different motilities exhibited by singlet T. gondii and by infected cells transporting intracellular parasites. To investigate the dissemination of singlet tachyzoites compared to infected cells harboring vacuoles of multiple intracellular parasites, we followed these motility events over time. Of note, only parasites that exhibited motility were tracked, and stationary tachyzoites or stationary infected cells were not included in this motility analysis. Singlet tachyzoites, although clearly moving, had confined trajectories that centered around a point, with frequent looping back over the existing path (Fig. 2A). In contrast, infected cells, which were identified as cells with vacuoles of at least two or more intracellular parasites, moved significantly farther from their point of origin regardless of travel speed (Fig. 2B). The confined movements of individual tachyzoites corresponded with relatively low speeds, as these tachyzoites moved at 1.37 ± 1.28 μm/min on average (Fig. 2C). Among the infected cells transporting intracellular vacuoles of T. gondii, we identified two distinct categories of motile cells: infected cells moving at either low (0.50 ± 0.24 μm/min) or high (6.30 ± 3.09 μm/min) speeds (Fig. 2C). Notably, the fast-moving infected cells harboring multiple tachyzoites moved significantly faster than the singlet tachyzoites (Fig. 2C). Consistent with their relatively high speeds, these fast-moving infected cells had greater total path lengths and maximal displacements compared to both the slow-moving infected cells and the singlet tachyzoites (Fig. 2D, E). Interestingly, although a subset of singlet tachyzoites (20%) traveled at slightly higher speeds (Fig. 2C) and had greater path lengths (Fig. 2D), they did not travel farther from their point of origin, i.e., they did not have an increase in maximal displacement (Fig. 2E). These data indicate that the volume of space within which extracellular tachyzoites move is relatively confined, whereas infected cells show greater displacement and spread within the tissue.

### During acute infection, T. gondii infects infiltrating peripheral immune cells and resident microglia in the brain.

Next, we determined the degree to which brain resident cells or infiltrating immune cells could transport intracellular T. gondii through the brain. Most brain resident cells have limited motility. However, microglia, which are macrophage-like phagocytic cells of the brain that play a key role in shaping brain development (30), dynamically extend and retract their branched processes to survey the brain parenchyma for injury, damage, and debris (31–33). During acute T. gondii infection of mice, there is also a marked increase in peripheral immune cell infiltration in the brain (28). Based on the relatively high speeds of the infected cells harboring intracellular parasites and their notable flexibility/malleability as they moved through a dense tissue environment, we hypothesized that infiltrating immune cells, or perhaps microglia, may be capable of moving through the brain in this manner.

**FIG 3:**
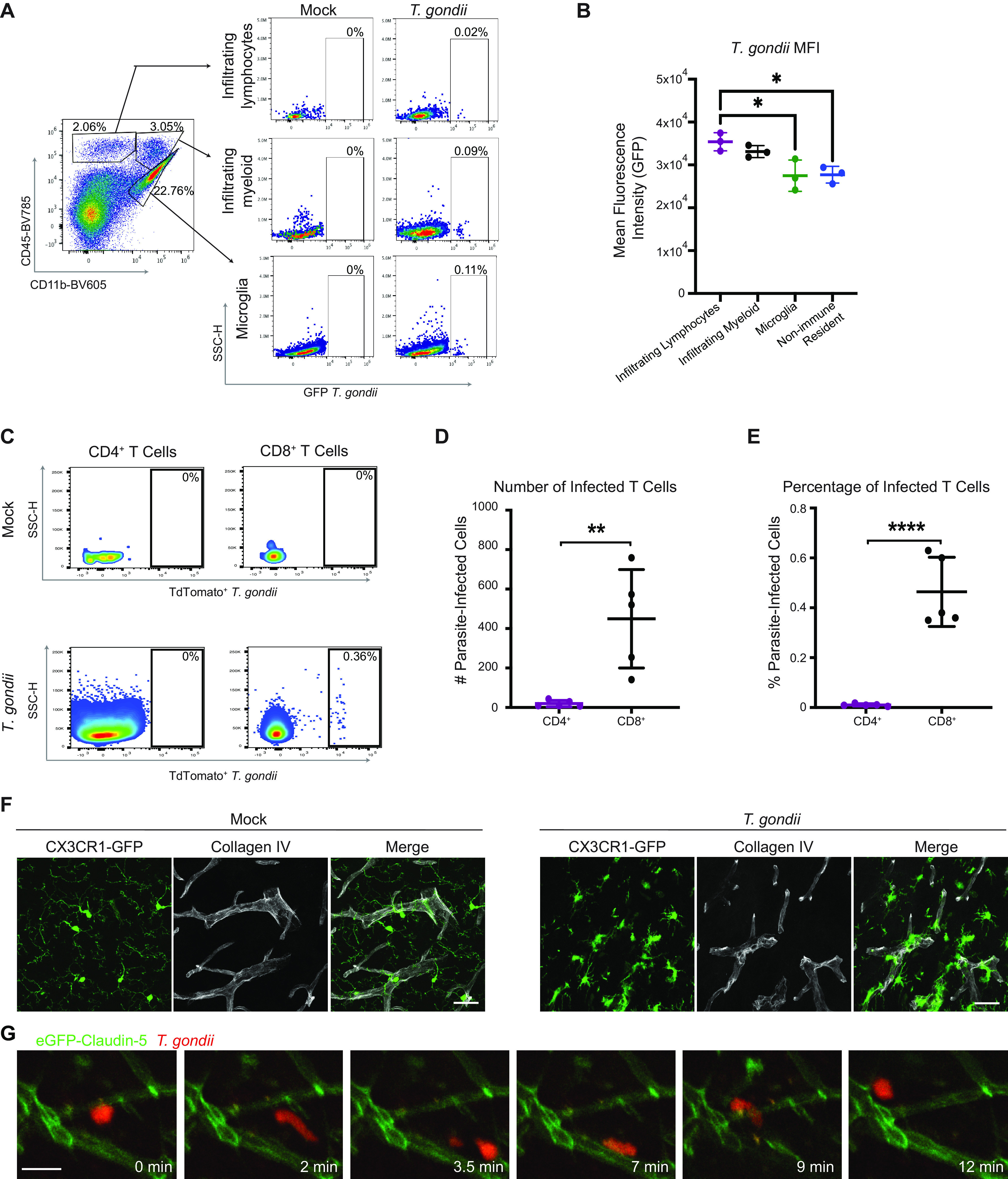
T. gondii infection of microglia and infiltrating immune cells during acute infection. (A) Single cells were isolated from the brains of C57BL/6 mice injected with PBS (mock condition) or infected with 200 GFP-expressing type II T. gondii tachyzoites at 8 dpi. Shown are representative flow cytometry plots of immune cell populations based on anti-CD11b and anti-CD45 antibody staining from an infected mouse (left plot) and the cells within each population. The percentage of each cell population that is infected (GFP^+^) is shown on the right. SSC-H, side scatter. (B) Quantification of the mean fluorescence intensity (MFI) of GFP in infected cell populations. (C) Immune cells were isolated from the brains of C57BL/6 mice injected with PBS (mock condition) or infected with 10^4^ tdTomato-expressing type II T. gondii tachyzoites at 14 dpi. Shown are representative flow cytometry plots of immune cell populations based on anti-CD4 (left plots) or anti-CD8 (right plots) antibody staining from a PBS-injected (top plots) or T. gondii*-*infected mouse (bottom plots). The cells within each population were gated on tdTomato^+^ parasite-infected cells, and the percentage of each cell population that is infected is shown. (D and E) Average number (D) and percentage (E) of infected CD4^+^ or CD8^+^ cells. *n* = 5 mice. **, *P* < 0.01; ****, *P* < 0.0001 by Student’s *t* test. (F) Brain sections from mock or T. gondii*-*infected CX3CR1-GFP mice were stained with anti-collagen IV antibody to delineate the blood vessels and imaged by confocal microscopy at a magnification of ×63. Scale bars, 30 μm. (G) Two-photon imaging was conducted through a cranial window in eGFP-claudin-5 mice infected with tdTomato-expressing T. gondii. A large vacuole (red) was tracked moving along vessels (green) in the brain during 12 min of imaging. Scale bar: 20 μm. Imaging was conducted at 8 dpi.

Flow cytometry on brain homogenates from wild-type C57BL/6 mice that were infected with GFP-expressing T. gondii was performed to determine the identity of infected cells in the brain at this acute time point. We gated cells based on their surface expression of CD45 and CD11b to identify infiltrating lymphocytes (CD11b^−^ CD45^hi^), infiltrating myeloid cells (CD11b^+^ CD45^hi^), and microglia (CD11b^+^ CD45^lo^), with the remaining brain resident cells identified as CD45^−^ cells. In mock-treated mice that were injected with phosphate-buffered saline (PBS), we did not detect GFP^+^ cells in the brain by flow cytometry in any of the cell populations (Fig. 3A). In the T. gondii*-*infected mice at 8 dpi, we detected GFP^+^ cells in each of the populations, with the majority of events in the CD45^−^ brain resident population (Fig. S2). Infected CD45^+^ cells were also detected, and these cells included microglia and infiltrating myeloid cells and lymphocytes (Fig. 3A). By calculating the mean fluorescence intensity (MFI) of the GFP^+^ events in each of the CD45^+^ populations, we found that the GFP expression was higher in immune cells infiltrating the brain than in the microglia or resident brain cells (Fig. 3B). These data suggest either that these cells are more frequently superinfected, with more invasion events occurring on a per-cell basis, or that the parasites replicated to a greater extent in these cells. Notably, we observed higher numbers and frequencies of infected CD8^+^ T cells than CD4^+^ T cells (Fig. 3C to E), indicating that the infected T cells were predominantly CD8^+^ T cells. We often detected a lower burden of parasite-infected cells by flow cytometry than would be suggested by conducting PCR for the parasite-specific B1 gene in brain homogenates from infected mice (Fig. S1B). This difference may be due to the extensive processing of the brain that is required to obtain single cells for flow cytometry analysis, which can result in the lysis, and therefore loss, of some infected cells. In addition, our brain cell isolation protocol does not enrich for nonhematopoietic cells, which account for the majority of infected cells in the brain.

10.1128/mbio.02838-22.9 FIG S2 Parasite-infected cells in the brains of T. gondii-infected mice. Immune and brain-resident cell populations in the brains of mice infected with GFP-expressing T. gondii were examined by flow cytometry and gated using CD45 and CD11b as in Fig. 3A. The percentage of GFP^+^ cells in each cell population is plotted. *n* = 3 mice per group. *, *P* < 0.05; ****, *P* < 0.0001 evaluated by one-way ANOVA. Download FIG S2, DOCX file, 0.1 MB. Copyright © 2022 Schneider et al. 2022 Schneider et al. https://creativecommons.org/licenses/by/4.0/ This content is distributed under the terms of the Creative Commons Attribution 4.0 International license.

To examine microglia during T. gondii infection, we utilized transgenic mice that express GFP under the control of the *CX3CR1* promoter (CX3CR1-GFP mice) (34). CX3CR1 is a chemokine receptor for CX3CL1 (fractalkine) (35) and is highly expressed on the surface of microglia and expressed at lower levels on some infiltrating monocytes and lymphocytes (36). By examining fixed brain tissue sections from CX3CR1-GFP mice during T. gondii infection, we detected microglia surrounding and in contact with the blood vessels in the brain, in contrast to their tiled localization in the parenchyma of mock-infected mice (Fig. 3F). The juxtavascular localization of microglia observed in infected mice has been described during development of the mouse and human brain (37), as well as in instances of vascular injury (38). In our intravital imaging, we noted that fast-moving cells harboring intracellular parasites frequently traveled along the vasculature, as seen by turns made >45° in the track trajectory that resulted in the vacuole traveling parallel to a vessel (Fig. 3G and Movie S4). The juxtavascular location of the microglia and the fact that they harbored parasites during acute infection suggested that they might be capable of actively shuttling T. gondii through the brain.

10.1128/mbio.02838-22.4 MOVIE S4 Motility of a T. gondii-infected cell in the brain at 8 dpi. eGFP-claudin-5 mice were infected with tdTomato-expressing T. gondii and 2-photon imaging through a cranial window was conducted at 8 dpi. An infected cell with a large vacuole containing multiple tdTomato-expressing parasites was imaged moving through the brain parenchyma along the vasculature. The cell was imaged for 37 min at 1 frame every 0.5 to 1 min. This video corresponds to the filmstrip in Fig. 3G (filmstrip timestamps of 0 to 12 min correspond with 19 to 31 min of the video). The field of view shifts twice to follow the vacuole as it is transported within an infected cell in the brain. Download Movie S4, MOV file, 13.0 MB. Copyright © 2022 Schneider et al. 2022 Schneider et al. https://creativecommons.org/licenses/by/4.0/ This content is distributed under the terms of the Creative Commons Attribution 4.0 International license.

### T. gondii-infected microglia have limited motility.

**FIG 4:**
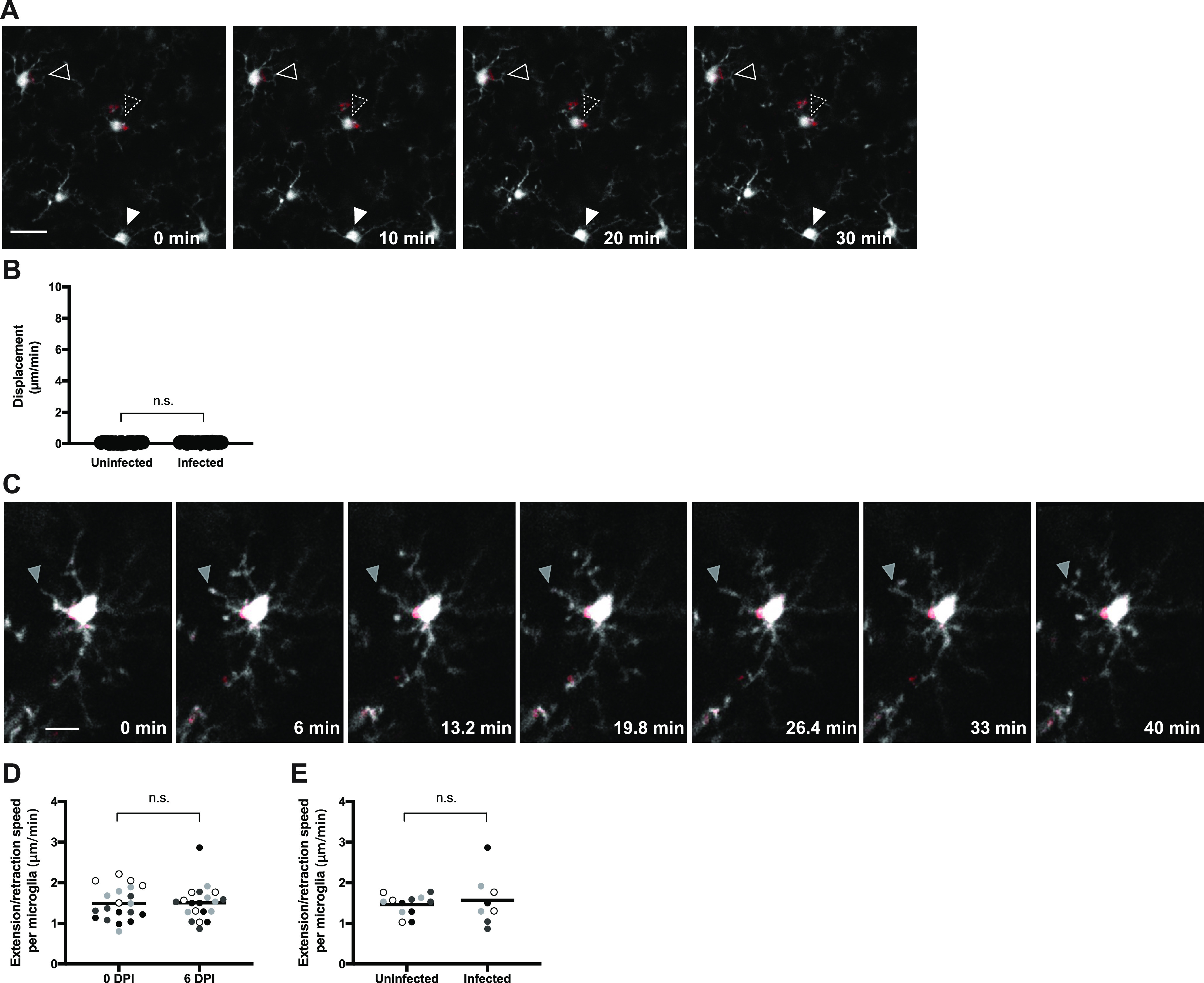
Microglia exhibit limited motility during acute infection. Microglia were imaged for 30 to 60 min via time-lapse 2-photon microscopy in CX3CR1-GFP mice infected with tdTomato^+^ T. gondii. (A) Infected (two open arrowheads) and uninfected microglia (filled arrowhead) were imaged at 6 dpi. CX3CR1-GFP is shown in white and T. gondii are in red. Scale bar: 20 μm. (B) Displacement of uninfected or infected CX3CR1-GFP^+^ microglia in mice at 6 dpi. Infected microglia were identified as those with vacuoles of intracellular parasites. *n*_uninfected_ = 23 cells; *n*_infected_ = 25 cells. (C) The movement of an individual microglial process (filled gray arrow) was imaged over time. CX3CR1-GFP is shown in white and T. gondii are in red. Scale bar: 10 μm. (D and E) Extension and retraction of all processes from each microglial cell were tracked and averaged to obtain an extension/retraction speed of processes per microglial cell. Movement of microglial processes was compared in mice at 0 dpi and 6 dpi (D) and in uninfected and infected microglia at 6 dpi (E). Each dot represents an individual cell, and the dots in different shades of gray represent cells from four different mice. In panel D, *n* = 20 microglia per time point, and in panel E, *n*_uninfected_ = 12 cells and *n*_infected_ = 8 cells. n.s., not significant, Student’s *t* test.

To investigate the possibility of microglia trafficking T. gondii in the brain, we implanted cranial windows on CX3CR1-GFP mice prior to infection with tdTomato-expressing T. gondii. Two-photon imaging was performed through the windows for 30 to 60 min at baseline (0 dpi, prior to infection) and at 6 dpi. Unlike at baseline, at 6 dpi, microglia infected with tdTomato^+^ T. gondii were visible in the brain (Fig. 4A). By tracking the cell body/soma for all CX3CR1-GFP^+^ microglia in the imaging fields of view (FOVs) at 6 dpi, we found that the microglial cell bodies were essentially stationary, and neither uninfected bystander microglia nor parasite-infected microglia exhibited motility (Fig. 4B). Indeed, in imaging infected CX3CR1-GFP^+^ microglia in multiple FOVs from four different mice, we never observed microglia shuttling parasites through the brain; rather, the cell bodies of infected microglia remained fixed in their starting positions for the duration of the imaging sessions (Fig. 4A to C).

Although we did not detect significant movement of the microglia cell body/soma during imaging, these cells also have processes that perform continuous searching, both in homeostasis and during injury (31, 32). Among the infected microglia that we imaged, T. gondii organisms were located within the soma 96% of the time, and parasites were rarely found in the microglial projections (and in those few cases, the parasites remained stationary over 60 min of imaging). To evaluate if T. gondii infection altered the dynamics of microglial projections, we measured the speed of extension and retraction of individual microglial processes at baseline and at 6 dpi (Fig. 4C and D and Movie S5). We found that the processes often had a bulbous endings, as previously described (31, 32), and moved at a mean speed of 1.49 ± 0.35 μm/min at baseline (Fig. 4D), which is consistent with prior reports of extension/retraction of resting microglia (31, 32). This movement of microglial processes was not significantly different in mice between baseline and 6 dpi (Fig. 4D) and was independent of whether the microglia harbored a parasite (Fig. 4E). Taken together, these data indicate that in our model, infected microglia did not appear to be a major vehicle for T. gondii dissemination and also did not exhibit infection-induced hypermotility during acute infection.

10.1128/mbio.02838-22.5 MOVIE S5 Stationary T. gondii*-*infected microglia with dynamic extension-retraction of processes. CX3CR1-GFP mice were infected with tdTomato-expressing T. gondii. Two-photon imaging through a cranial window was conducted at 6 dpi. An infected CX3CR1-GFP^+^ microglia (white) with a vacuole of T. gondii (red) was imaged for 40 min at 3 frames every 1 min. This movie corresponds with the filmstrip in Fig. 4C. Download Movie S5, AVI file, 1.2 MB. Copyright © 2022 Schneider et al. 2022 Schneider et al. https://creativecommons.org/licenses/by/4.0/ This content is distributed under the terms of the Creative Commons Attribution 4.0 International license.

### Infiltrating peripheral immune cells can shuttle T. gondii within the brain.

**FIG 5:**
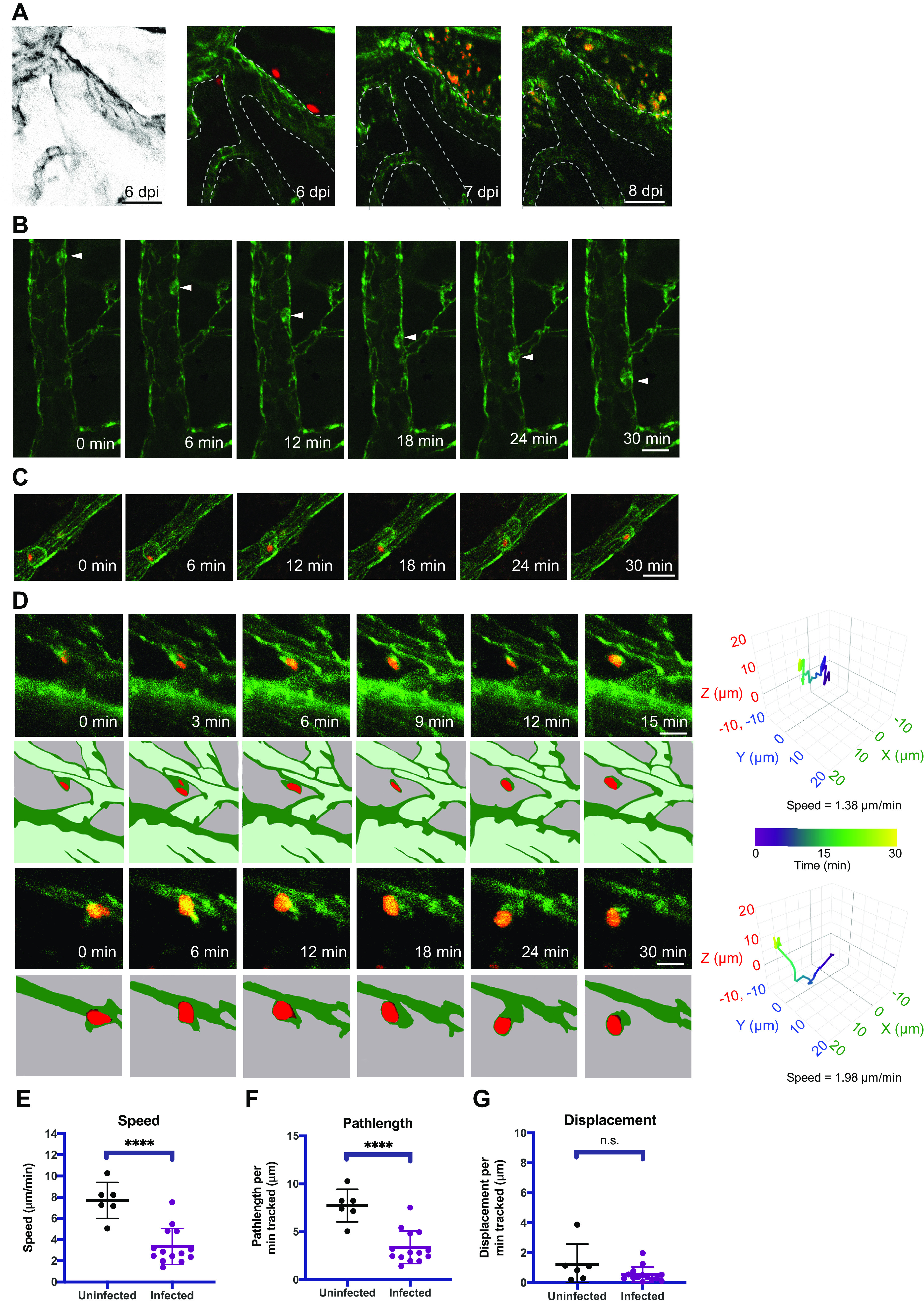
Infiltrating peripheral immune cells traffic T. gondii parasites in the brain. Infiltrating immune cells were imaged via time-lapse 2-photon microscopy in eGFP-claudin-5 mice infected with tdTomato^+^ T. gondii by injecting the mice i.v. with anti-CD45-AF488 antibodies. (A) Longitudinal imaging of the same FOV from 6 to 8 dpi reveals intact vacuoles of parasites at 6 dpi followed by massive expansion in parasite numbers in the brain during acute infection. (B) A CD45^+^ peripheral immune cell was tracked migrating along the vessel lumen (white arrowhead) over 30 min. Anti-CD45-AF488 signal on immune cells is distinguishable from eGFP-claudin-5 signal in the blood vessels. (C) A T. gondii*-*infected CD45^+^ cell migrating in the vessel lumen was tracked over 30 min of imaging. (D) Infected CD45^+^ cells from mice at 8 dpi were tracked in the brain. Motility plots for each cell are shown at the right in 3D. Colors in the traces reflect the length of time of imaging for each cell. (E to G) Average cell speed (E), path length (F), and displacement (G) of uninfected and infected CD45^+^ cells in the brain were recorded and plotted. *n* = 6 uninfected CD45^+^ cells (E to G) and *n* = 14 infected CD45^+^ cells. Imaging shown in panels A to D was done on days 5 to 8 postinfection and reflect data from 3 infected mice. Scale bars: 50 μm (A) or 20 μm (B to D). ****, *P* < 0.0001 by Student’s *t* test.

We next examined a potential role for peripheral immune cells as a vehicle for T. gondii in the brain. To address this possibility, cranial windows were installed in eGFP-claudin-5 mice, and the mice were infected with tdTomato-expressing T. gondii. To fluorescently label circulating hematopoietic immune cells, we injected Alexa Fluor 488 (AF488)-conjugated anti-CD45 antibodies intravenously (i.v.) 24 h prior to each 2-photon imaging session. Importantly, at 6 dpi the injection of anti-CD45-AF488 during infection did not result in labeling of microglia or macrophages within the brain (Fig. 5A, left), suggesting that the antibody did not cross the BBB during these acute time points. This strategy enabled us to visualize individual T. gondii vacuoles located adjacent to blood vessels and large clusters of parasites amid CD45^+^ cells within the brain parenchyma during longitudinal imaging of the same FOV from 6 to 8 dpi (Fig. 5A). Although both the eGFP-claudin-5 and the CD45^+^ cells fluoresced green, we could clearly distinguish the vasculature from the peripheral CD45^+^ cells within the vessel lumen when the anti-CD45-labeled cells interacted with the blood-brain barrier (Fig. 5B). Using this labeling approach, we detected T. gondii*-*infected immune cells migrating in the cerebral vasculature (Fig. 5C) and captured what appeared to be a rare cell division event (Fig. 5C, 12- to 30-min timestamps, and Movie S6). Most notably, we observed infected CD45^+^ cells traveling extravascularly (Fig. 5D), consistent with our prior imaging showing infected cells shuttling T. gondii within the brain. Motility maps of individual infected cells demonstrated the nonlinear paths of these infected CD45^+^ cells in the brain (Fig. 5D, right). Finally, by quantifying the motility of CD45^+^ uninfected or infected cells, we found that uninfected CD45^+^ cells traveled within the brain at 7.70 ± 1.71 μm/min on average, whereas infected CD45^+^ cells traveled at 3.35 ± 1.70 μm/min on average (Fig. 5E). This travel speed is slightly lower than that of the fast-moving infected cells detected in the eGFP-claudin-5 mice, which may be due to the fact that these cells were coated with fluorescent anti-CD45 antibody, potentially influencing their migration. In addition, this analysis includes the speeds of both slow- and fast-moving infected cells. The average path length of infected CD45^+^ cells was shorter than that of uninfected CD45^+^ cells (Fig. 5F); however, their maximal displacement was not different (Fig. 5G). These data indicate that T. gondii infection of CD45^+^ cells did not result in increased motility speeds compared to uninfected CD45^+^ cells within the brain. However, the findings support a model whereby infiltrating immune cells from the periphery can shuttle T. gondii tachyzoites within the brain and increase their dissemination compared to extracellular T. gondii parasites migrating on their own.

10.1128/mbio.02838-22.6 MOVIE S6 T. gondii-infected CD45^+^ immune cell migrating within a blood vessel in the brain. eGFP-claudin-5 mice were infected with tdTomato-expressing T. gondii (red) and injected with AF488-conjugated anti-CD45 antibodies. Two-photon imaging through a cranial window was conducted at 8 dpi. An infected CD45^+^ cell (green) is migrating within the lumen of a blood vessel in the brain. Imaging was conducted for 30 min at 1 frame per minute. This movie corresponds with the filmstrip in Fig. 5C. Download Movie S6, AVI file, 0.4 MB. Copyright © 2022 Schneider et al. 2022 Schneider et al. https://creativecommons.org/licenses/by/4.0/ This content is distributed under the terms of the Creative Commons Attribution 4.0 International license.

## DISCUSSION

T. gondii infection of the CNS can result in substantial pathology in immunocompromised individuals. Recent evidence has emerged for endothelial cells as a key portal for T. gondii infection at the BBB and for entry into the brain (23) and transit across cortical capillaries (35); however, little is known about how the parasites arrive at the BBB or spread through the brain during infection. We investigated the motility of T. gondii*-*infected cells in the cerebral vasculature and in the brains of living mice in comparison with singlet extracellular T. gondii. In two-dimensional (2D) *in vitro* systems, T. gondii tachyzoites are known to exhibit circular and helical gliding (39), whereas *in vivo*, their movements have the appearance of corkscrew-like motility (29). Consistent with a previous report of T. gondii motility in the mouse earflap (29), we also observed singlet tachyzoites moving in a corkscrew-like manner through the brain parenchyma. Interestingly, however, in the brain, the motility of singlet T. gondii parasites largely resulted in relatively constrained movement without substantial displacement from the starting point of the imaging session. In contrast, intracellular T. gondii parasites were transported at rapid speeds (6.30 ± 3.09 μm/min) through the brain parenchyma during acute parasite infection and dissemination. In the brain, the trafficking of T. gondii in infected cells was likely due to the infection of infiltrating immune cells, as infected resident microglia were not observed to have motility during long-term imaging sessions.

Previous studies on the motility of T. gondii-infected leukocytes reported travel speeds similar to those we detected for infected cells in the brain. During infection with T. gondii *in vitro*, monocytes and dendritic cells (DCs) become hypermotile (17, 19), and DC hypermotility is mediated by a parasite protein, T. gondii 14-3-3 (Tg14-3-3) (40). Likewise, the parasite-secreted effector protein TgWIP enhances DC hypermotility and parasite spread from the peritoneal cavity to distal organs (25). *In vivo*, however, CD11c^+^ cells in the CNS (likely a mixture of DCs and microglia) interact with T cells, but they are not observed to travel greater than 2 μm/min during chronic infection (41). Though T cells represented only a small proportion of infiltrating cells during our acute time points, it is possible that the actively transported parasite vacuoles we observed were within T cells in the CNS: imaging of brain explants from mice has revealed T cells traveling at a velocity of 6.35 μm/min and identified T cells trafficking parasites during chronic infection (39, 40). Indeed, T. gondii infection of T cells is frequently observed during intimate contacts with infected antigen-presenting cells in peripheral lymph nodes and in the brain (42). NK cells also exhibit hypermotility in the lymph nodes during T. gondii infection, with velocities averaging >10 μm/min (21); however, we rarely find NK cells in the CNS during acute T. gondii infection (28), and NK cells do not result in spread to the CNS when adoptively transferred (43). Monocytes were also found to be infected by *T. gondii* at these acute time points. Although methods exist for the depletion of myeloid cells through genetic or chemical means, it is difficult to utilize these strategies to examine the contribution of myeloid cells to parasite spread through the brain, since the loss of myeloid cells, particularly Ly6C^+^ monocytes, is lethal to mice in acute T. gondii infection and causes substantial neurological pathology in chronic infection (44, 45).

We considered the possibility of microglia as a potential host cell vehicle for T. gondii in the brain, given their juxtavascular localization in infected mice and their potential for migration (46). Moreover, primary mouse microglia infected with T. gondii were found to exhibit hypermotility *in vitro* (18). During acute infection *in vivo*, however, we did not observe significant microglial movement in the intact brain. It is possible that differences in cytokine exposure, physical confinement by other cells in the brain, or additional factors explain the difference between the *in vitro* and *in vivo* observations. Since we never observed infected microglia moving rapidly through the brain, these cells did not appear to contribute in a substantive way to T. gondii dissemination in our model. However, we cannot rule out that these events may occur outside our imaging window or that microglia may transport T. gondii, perhaps at other time points, as the brain environment changes in response to the infection.

One persistent question that is addressed by the current studies is the degree to which T. gondii increases the motility of cells that it infects in the brain. In the periphery, *ex vivo* imaging studies have demonstrated that T. gondii infection of NK cells in the lymph nodes increases the motility of these cells (21) and that infected neutrophils in the small intestine can cross the epithelial layer to disseminate within the lumen (47). Infected CX3CR1^+^ myeloid cells exhibit increased tissue migration in the spleen in a process dependent on the parasite kinase ROP17 (22), which is involved in translocating dense granule proteins across the parasitophorous vacuole membrane (48). In contrast, in our live-imaging study, neither the infection of infiltrating CD45^+^ cells nor the infection of brain resident microglia increased the speed or distance traveled by these cells, suggesting that infection-induced hypermotility is not a prominent feature of CNS infection. This finding may be a function of the dense cellular network of brain tissue and the fact that T. gondii has a rigid cytoskeleton, which may hinder the motility of infected cells carrying a large cargo of parasites. However, in comparing the motility of T. gondii*-*infected cells to that of extracellular tachyzoites, the infection of motile immune cells was associated with significantly increased parasite dissemination.

T. gondii establishes a chronic infection in the CNS, with an enrichment of parasites noted in the cortex compared to other regions in the brain (49). The unprecedented speed and displacement of the rapidly transported T. gondii would facilitate coverage of nearly 1 cm of distance per day, a remarkable distance. Given the robust immune response observed surrounding clusters of T. gondii tachyzoites in the brain, the ability of the parasites to infect motile cells and move away from sites of immune-mediated clearance may prove advantageous as a strategy of immune evasion that contributes to T. gondii survival within the brain. Parasite latency is achieved over a period of weeks, easily enabling parasites within motile infected cells to become highly dispersed from their starting position. In chronic infection, the widely distributed cyst load observed (50) may be due to focal infiltration in the brain at a few unique sites, with rapid transport of parasites throughout the brain as the infection transitions into latency. Enhanced neuroimaging of these intriguing dissemination events will likely yield more insight into the cellular mechanisms that underlie T. gondii infection and neuropathogenesis.

## MATERIALS AND METHODS

### Mice and infection experiments.

All mouse experiments were approved by and performed in accordance with guidance from the IACUC committee at the University of California, Irvine (UCI). Male and female mice were bred on the C57BL/6 background at UCI and used for experiments between 2 and 8 months of age. Heterozygous eGFP-claudin-5 and wild-type (WT) mice were generated by breeding heterozygous eGFP-claudin-5 mice to WT mice and by genotyping using PCR. CX3CR1-GFP mice were generously provided by Karina Cramer and Kim Green at UCI from their breeding colonies. Mice were provided food and water *ad libitum* and housed with corn cob bedding. Mice were infected with T. gondii tachyzoites by intraperitoneal injection as described previously (28). Briefly, type II GFP-expressing Prugniaud (51) or tdTomato-expressing Prugniaud tachyzoites were cultured in monolayers of human foreskin fibroblasts, syringe lysed, filtered, and washed before injecting into mice. A total of 1 × 10^3^ to 5 × 10^4^ tachyzoites in PBS were injected per mouse.

### Cranial window implantation.

Mice underwent surgery as previously described in detail (28). Briefly, a 3D printed plastic headplate was affixed to the mouse skull using Vetbond (3M) and acrylic resin Ortho-Jet BCA (Lang Dental). The headplate was then attached to a stereotactic frame and a 3-mm craniotomy was centered over the right hemisphere between bregma and lambda. An additional 1 mm of the outer skull region was thinned. A 4-mm glass coverslip (World Precision Instruments, Sarasota, FL) was placed on the exposed brain and, under pressure against the thinned bone, glued using Vetbond and acrylic resin. Mice were given carprofen (10 mg/kg of body weight subcutaneously [s.c.]) for a minimum of 7 days and monitored for signs of postoperative bleeding. Mice with postoperative bleeding were excluded from imaging. Mice were infected a minimum of 24 h after the final dose of carprofen.

### *In vivo* 2-photon microscopy.

We used a resonant 2-photon microscope (Neurolabware) equipped with a water immersion Olympus objective (20×, 1.0 numerical aperture [NA]). The reporters (GFP and tdTomato) were simultaneously excited using a femtosecond laser set to 900 to 920 nm (Mai Tai HP; Spectra-Physics, Santa Clara, CA). Emissions were filtered using a 510/84-nm and 607/70-nm BrightLine bandpass filter (Semrock, Rochester, NY), and images were gathered using Scanbox acquisition software (Scanbox, Los Angeles, CA). For imaging of microglia, we used an electrically tunable lens (Optotune) to rapidly toggle (10 Hz) between 10 focal planes (3 to 10 μm apart) every second. For parasite and CD45 cell imaging, a z-stack was acquired by imaging each plane for 10 to 20 frames. The same volume was then reimaged every 30 to 60 s. FIJI (52) was used to bin and motion correct (HyperStackReg05) recordings. Imaris (Bitplane) was used to quantify motility.

### Confocal microscopy.

Brain hemispheres were removed from PBS-perfused mice, fixed in 4% paraformaldehyde (PFA) for 3 to 16 h, and cryopreserved in 30% sucrose until the tissue sank to the bottom of the tube. Brains were embedded in OCT freezing medium and sectioned into 25 μm-thick sagittal tissue sections for staining. Brain sections were stained with anti-IBA-1 (Dako, Japan) and either anti-CD31 (R&D Systems, Minneapolis, MN) or anti-collagen IV (EMD-Millipore, Germany). Sections were permeabilized for 1 h in permeabilization buffer (0.3% Triton X-100, 3% bovine serum albumin, 1× PBS) and incubated at room temperature for 16 to 48 h with primary antibodies diluted 1:500 in permeabilization buffer. Sections were washed with 1× PBS three times and incubated with secondary antibodies (Thermo Fisher) diluted in permeabilization buffer for 2 h to overnight. Sections were washed and mounted (with or without 4′,6-diamidino-2-phenylindole [DAPI]) for imaging on an SP8 confocal microscope (Leica).

### Flow cytometry.

Single cells were isolated from one brain hemisphere from PBS-perfused mice as previously described (28). Briefly, each brain hemisphere was minced with scissors and incubated in 2 U/mL of Dispase II enzyme (Roche Applied Science) resuspended in HEPES buffered saline for 1 h at 37°C. Cells were triturated 20 times with a 10-mL serological pipette and passed through a 70-μm mesh filter. Myelin was removed with a 70:30 Percoll gradient (GE Healthcare). Cells were resuspended in FACS buffer (1× PBS plus 3% fetal bovine serum [FBS]) with 10% TrueStain FcX (Biolegend, San Diego, CA) to block nonspecific antibody binding to Fc receptors. Cells were stained with directly conjugated antibodies against the cell surface marker CD4 (from BD) or CD11b, CD45, CD3, or CD8a (all from Biolegend) for 30 min and then washed with FACS buffer and fixed in 2% paraformaldehyde. Stained cells were run on a Novocyte flow cytometer (ACEA, San Diego, CA). Data were analyzed using FlowJo software (Treestar, Ashland, OR).

### Cell tracking and mapping.

Single parasite tachyzoites, parasite vacuoles, and microglia soma were rendered as surfaces and tracked using IMARIS software (Bitplane, Zurich, Switzerland) to determine path length, displacement, positions, and velocity. 3D flower plots were generated in R using the Plotly package (53). Microglial projection surveillance activity was quantified using the filament tracer function in IMARIS to trace extension and retraction of microglial processes over time. Lengths of filaments were compared over time to calculate speeds.
