# Supplementary material for: Toxoplasma gondii Dissemination in the Brain Is Facilitated by Infiltrating Peripheral Immune Cells
Source: mBio. 2022 Nov 29;13(6):e02838-22. doi: 10.1128/mbio.02838-22 (PMC9765297; doi:10.1128/mbio.02838-22)
Supplement: FIG S1 [file mbio.02838-22-s0007.docx]

**Supplementary Information**

**(Schneider *et al.*)**

**Figure S1**
