# Supplementary material for: Toxoplasma gondii Dissemination in the Brain Is Facilitated by Infiltrating Peripheral Immune Cells
Source: mBio. 2022 Nov 29;13(6):e02838-22. doi: 10.1128/mbio.02838-22 (PMC9765297; doi:10.1128/mbio.02838-22)
Supplement: TEXT S1 [file mbio.02838-22-s0009.docx]

**Supplementary Information**

**(Schneider *et al.*)**

**Supplemental Methods**

**Parasite Quantification**

To determine the parasite burden in the brains of infected mice, the Qiagen Blood and Tissue kit was used to isolate DNA from 10 mg of homogenized brain tissue. The *T. gondii* B1 gene was amplified using qPCR with each sample run in triplicate using a Bio-Rad iCycler PCR system and the Bio-Rad iTaq Universal SYBR Green Supermix. The primer pairs for B1, CAGATGTGCTAAAGGCGTCA (sense), and GCCCTAGACAGACAGCGAAC (anti-sense), were synthesized by Integrated DNA technologies. Parasite concentrations (parasites/mg) were determined by referencing a standard curve of B1 data generated from known quantities of parasites as previously published (1). qPCR outputs were analyzed using the threshold cycle method previously published (2). Negative controls consisting of replacing the DNA template with water, and these samples were confirmed to have no amplification.

1. J. L. Burg, C. M. Grover, P. Pouletty, J. C. Boothroyd, Direct and sensitive detection of a pathogenic protozoan, Toxoplasma gondii, by polymerase chain reaction. *J Clin Microbiol* **27**, 1787-1792 (1989).

2. K. J. Livak, T. D. Schmittgen, Analysis of relative gene expression data using real-time quantitative PCR and the 2(-Delta Delta C(T)) Method. *Methods* **25**, 402-408 (2001).
